# Study on Hardness of Heat-Treated CoCrMo Alloy Recycled by Electron Beam Melting

**DOI:** 10.3390/ma16072634

**Published:** 2023-03-26

**Authors:** Katia Vutova, Vladislava Stefanova, Martin Markov, Vania Vassileva

**Affiliations:** 1 Institute of Electronics, Bulgarian Academy of Sciences, 1784 Sofia, Bulgaria; 2 Department of Metallurgy of Non-Ferrous Metals and Semiconductors Technologies, University of Chemical Technology and Metallurgy, 1756 Sofia, Bulgaria

**Keywords:** CoCrMo alloy, electron beam melting and refining, heat treatment, hardness

## Abstract

The hardness of heat (thermally) treated CoCrMo ingots, recycled by electron beam melting and refining (EBMR) of a technogenic CoCrMo material (waste from the dental technology) under different process conditions (temperature and residence time) is examined. The heat treatment consists of two-step heating up to temperatures of 423 K and 1343 K and retention times of 40 and 60 min, respectively. The influence of various loads (0.98 N, 1.96 N, 2.94 N, 4.9 N, and 9.8 N) on the hardness of the CoCrMo alloy, recycled by EBMR, before and after heat treatment is studied. It has been found that regardless of the EBMR process conditions, the obtained samples after heat treatment have similar hardness values (between 494.2 HV and 505.9 HV) and they are significantly lower than the hardness of the specimens before the heat treatment. The highest hardness (600 HV) is measured in the alloy recycled at 1845 K refining temperature for 20 min. This is due to the smaller crystal structure of the resulting alloy and the higher cobalt content. The results obtained show that the heat treatment leads to considerable changes in the microstructure of the CoCrMo ingots recycled by EBMR. With the increase of the e-beam refining temperature, after the heat treatment, the grains’ size increases and the grains’ shape indicates an incomplete phase transition from γ-fcc to ε-hcp phase. This leads to a slight increase in the hardness of the alloy.

## 1. Introduction

Over the last decades, CoCrMo superalloys have found wide application in medicine for the manufacture of dental and medical (hip and knee joints, etc.) implants. This is due to their high resistance to wear and corrosion, good mechanical properties and biocompatibility with the human body [1].

The global market for CoCrMo alloys is constantly growing [2]. It is expected to reach $2.6 billion by 2030, and according to Compound Annual Growth Rate (CAGR) it will grow by 1.2% over the period 2021–2030. A key factor for this market growth is both the growing demand for dental and medical implants in developing countries, as well as the use of CoCrMo alloys in high-tech applications such as gas turbines, aircraft engines, etc.

CoCrMo alloys can be produced using a traditional melting and casting method and other alternative methods such as vacuum casting, laser-based powder bed fusion of metals (PBF-LB/M), computer-aided design/computer-aided manufacturing, laser sintering, etc. [3,4,5,6,7,8,9]. In recent years, electron beam melting [10,11,12] has been increasingly used in the production of surgical implants due to the possibility of easily obtaining the final form of the product.

The microstructure and mechanical properties of the CoCrMo alloys are strongly related to their chemical and phase composition, which can vary significantly depending on the method of their production, the cooling rate of the melt, the content of other alloying elements, etc. [7].

Usually, CoCr alloys contain two types of crystal structures that determine their physical, chemical and mechanical properties: a cobalt-rich face-centered cubic γ-fcc phase stable at high-temperature and a low-temperature cobalt-rich hexagonal close-packed ε-hcp phase [13,14,15].

The temperature of the γ-fcc to ε-hcp phase transformation can be changed due to the presence of other alloying elements in the alloy [1]. Elements such as Fe, Mn, Ni, Nb, and C decrease the transformation temperature, i.e., they are stabilizers of the γ-fcc phase, while Cr, Mo, W, and Si metals increase the transformation temperature, therefore, they are stabilizers for the ε-hcp phase. These transformations are closely related to the mechanical and chemical properties of the alloy. Suppression of the hcp ε-phase improves the mechanical and chemical properties of the alloy [1].

Cobalt is the main metal that determines the mechanical properties (hardness, strength and wear resistance, etc.) of the CoCrMo alloy, while chromium provides biocompatibility and corrosion resistance by forming a protective oxide (Cr_2_O_3_) layer [4].

The impact of the cobalt content on the hardness of different commercial brands of CoCrMo alloy is investigated in [16]. It is found that by increasing the cobalt content in the alloy from 59% to 64.6%, its hardness increases from 407 to 601 HV.

The influence of other alloying elements (such as Ni, W, and Mo) on the hardness of CoCrMo alloy is investigated in [17]. CoCrNi alloys have significantly lower hardness when compared to CoCrMo alloys. The hardness of the alloys decreases with increasing the nickel content and increases with increasing the chromium content.

CoCrNi, CoCrW, and CoCrMo alloys are characterized by different ratios of the γ-fcc and ε-hcp phases, which determine their mechanical properties [18]. Nickel stabilized the γ-fcc (42.3% phase content), while Mo and W stabilized the ε-hcp phase (78.8% and 64.5% phase content, respectively). The higher content of ε-hcp phases increases the hardness of the alloy and its value is highest forCoCrMo alloy (599 ± 9 HV) and lowest for CoCrNi alloy (296 ± 9 HV).

The conducted research [11,19] shows that the structure of CoCrMo alloy can be selectively transformed into a predominant ε or γ phase using a suitable heat treatment regime, which changes its mechanical properties, morphology and grain size.

A comparative analysis of the mechanical properties (tensile strength, micro hardness) before and after heat treatment at a temperature of 1493 K for 1, 2, and 4 h of a standard CoCrMo alloy (ASTM F75) produced by the selective laser melting (SLM) method is conducted in [20]. The authors observed an improvement in the tensile strength and a slight deterioration in the hardness of the alloy.

The impact of loads of 0.98 N, 1.96 N, 2.94 N, 4.9 N, and 9.8 N on the hardness of a CoCrMo alloy obtained by traditional casting and using the SLM method is examined in [21]. The hardness of the sample obtained after casting is 339 HV and after SLM it is 501 HV. Two of the samples obtained by SLM are thermally (heat) treated at 1473 K for 1 and 2 h with furnace and water cooling, respectively and the hardness of the alloy at the water cooling is ~486 HV, and that at the furnace cooling is ~455 HV. It is found that an increase in the load leads to a decrease in the hardness of the alloy and after a certain load it remains almost constant.

The influence of production parameters such as laser power, feed rate and scan speed on the hardness of the resulting alloy is examined in [22]. Further, the alloy is subjected to heat treatment—heating to 1473 K and retention for 30, 45, and 60 min and ageing at 1088 K and 1103 K for 2, 4, and 6 h, respectively. The highest hardness is obtained in samples of CoCrMo alloy produced at high laser power. The highest hardness (518 ± 69.41 HV) for a heat-treated alloy is obtained at 1473 K and 45 min time and ageing at 1103 K for 2 h.

The mechanical properties of several CoCrMo alloys produced under the electron beam melting process before and after heat treatment are tested in [12]. The heat treatment includes a hot isostatic pressing (HIP) and homogenizing processing. The measured Rockwell hardness is ~35 HRC (332 HV).

In our previous work [23], based on thermodynamic analysis and experimental research, the possibility of recycling the technogenic CoCrMo material (waste from the dental technology) by electron beam melting and refining (EBMR) was proven. The influence of the technological parameters on the change in the chemical composition of the alloy, on the behaviour of the elements (Fe, Mn, Si, W, and Nb) present in it, and on the microstructure of the resulting ingots was determined. The present study is a continuation of the research carried out so far and aims to study the hardness as one of the main mechanical properties of CoCrMo alloys, recycled by EBMR (under different processing conditions), before and after heat treatment (under the same conditions) and to determine the influence of different EBMR parameters (temperature and refining time) and heat treatment on the hardness of CoCrMo alloys.

## 2. Materials and Methods

**Figure 1:**
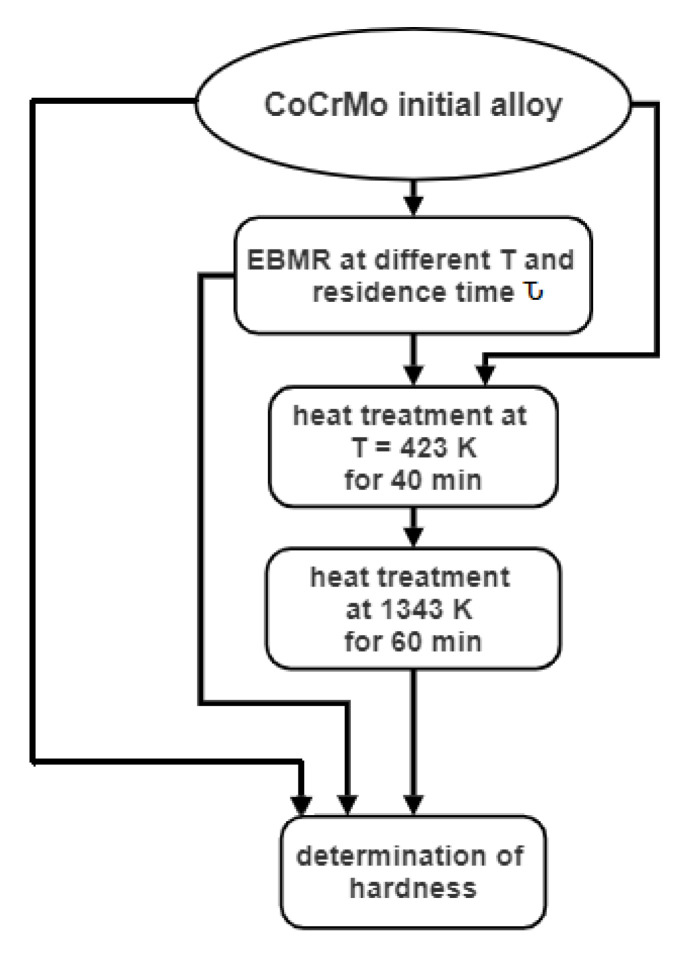
Flow chart of the processes involved in the processing of the studied material.

The microstructure and the hardness of the initial technogenic CoCrMo alloy and ingots obtained after recycling by EBMR, before and after heat treatment, are studied. Figure 1 shows the processes involved in the processing of the studied material. The electron beam melting of the technogenic material is carried out in a 60 kW electron beam furnace ELIT-60 (Leybold GmbH, Cologne, Germany), in the Institute of Electronics at the Bulgarian Academy of Sciences. The operating vacuum pressure is 1 × 10^−3^ Pa and the refined melt is formed in a water-cooled copper crucible with a movable bottom connected to a draw mechanism [24].

The melting parameters in the furnace (refining temperature T and residence time τ) and the chemical composition of the initial alloy and the resulting ingots are given in Table 1 (the standard deviation of the elements content is ~0.001%). The chemical composition of the studied specimens is determined by emission spectral analysis (UBI 1, Carl Zeiss, Jena, Germany).

The heat treatment of the initial alloy and the ingots obtained after recycling by EBMR includes the following stages. First, the samples are placed in a muffle furnace at a temperature of 294 K and heated to T = 423 K. At this temperature, the ingots are held for 40 min then they are heated to a temperature of 1343 K and retained for 60 min. The heating rate of the samples in the interval from 423 K to 1343 K is 9.2 grad/min. The heat treatment is carried out in an argon atmosphere.

The preparation of the specimens for the metallographic examination involves the standard procedure of grinding, polishing and etching. A Glyceregia reagent prepared from 15 mL HCl, 10 mL glycerol, and 5 mL HNO_3_ is used for etching [25]. The time for developing the microstructure of the studied samples is ~20 min.

A Leica DM2500 (Leica Microsystems GmbH, Wetzlar, Germany) light microscope with a digital camera Leica EC3 (Leica Microsystems GmbH, Germany) was used to study the macrostructure of the obtained specimens. The Leica LAS software (Leica Microsystems GmbH, Germany) was used for image processing.

The hardness of the studied samples is determined by the Vickers method using a Micro-Hardness Tester HV-1000 apparatus. The following loads are applied during the measurements: 0.98 N, 1.96 N, 2.94 N, 4.9 N, and 9.8 N.

## 3. Results and Discussion

### 3.1. Microstructures of CoCrMo Alloy Recycled by EBMR before and after Heat Treatment

Microscopic examinations have been performed to determine the influence of the heat treatment of CoCrMo alloy on the macro and microstructure. A light microscope which allows the examination of alloy morphology, macro and microstructure characteristics, determination of grains’ shape, inclusions’ distribution, presence of mechanical defects, etc. was used.

The phase transitions in CoCrMo alloys during cooling can be estimated using the binary phase diagram Co-Cr, calculated using Thermo-Calc and PBIN database [14,15,21].

**Figure 2:**
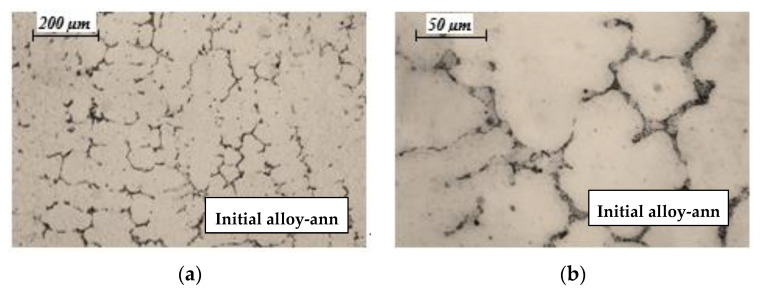
Optical microphotographs of initial CoCrMo alloy after heat treatment: (**a**) 100× magnification; (**b**) 400× magnification.

The microstructure of technogenic (initial) CoCrMo alloy after heat treatment—two-step heating to a temperature of 423 K and 1343 K and retention of 40 and 60 min, respectively, is shown in Figure 2.

It can be seen that after the heat treatment, the initial alloy has a metastable dendritic matrix, and chemical and intermetallic compounds are precipitated along its boundaries. The formation of a dendritic structure proceeds parallel to the solid/liquid border surface in the direction of heat removal. Large crystals (150–200 μm) are observed, surrounded by crystallized intermetallic melts and chemical compounds formed by the Fe, Mn, Si, Nb, and W elements present in the alloy.

**Figure 3:**
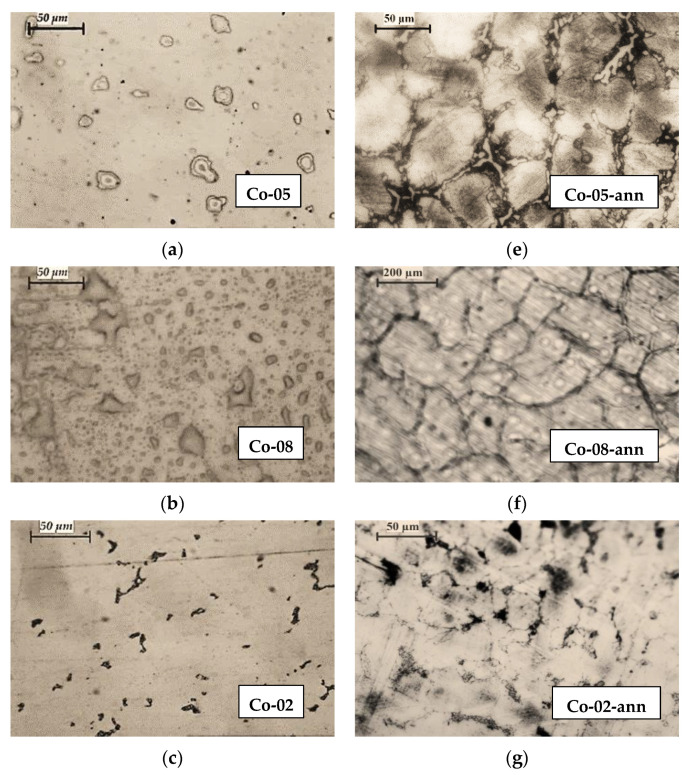

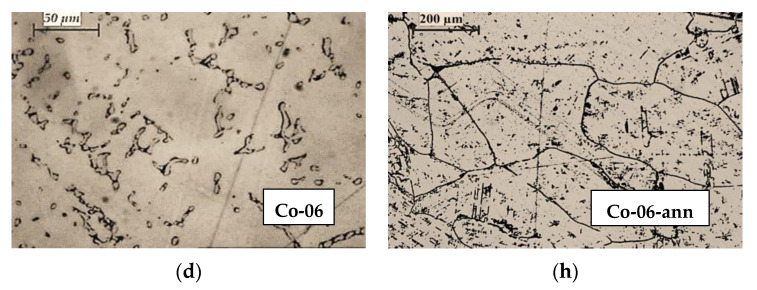
Microstructures of CoCrMo ingots recycled by EBMR: (**a**) Co-05; (**b**) Co-08; (**c**) Co-02; (**d**) Co-06 (400× magnification); and their microstructures after heat treatment: (**e**) Co-05-ann (400× magnification); (**f**) Co-08-ann (100× magnification); (**g**) Co-02-ann (400× magnification); (**h**) Co-06-ann (100× magnification).

In Figure 3, the microstructures of CoCrMo alloy samples after recycling by EBMR (Co-05, Co-08, Co-02, and Co-06) are compared to the microstructures of the same samples after two-step heat treatment (Co-05-ann, Co-08-ann, Co-2-ann, and Co-06-ann). Process parameters and chemical compositions of the specimens before and after EBMR are given in Table 1. It is obvious that the heat treatment process leads to significant changes in the microstructure of the CoCrMo alloys. Formation of large-sized grains is observed with inter-dendritic precipitates crystallizing along its borders. The shape of the grains indicates an incomplete phase transition from the high-temperature γ-phase with fcc cubic crystal lattice to the low-temperature ε-phase with a hexagonal crystal lattice.

Since the samples obtained under different EBMR conditions were heat-treated under the same conditions (Figure 1), it can be assumed that the structural changes observed on the microphotographs are closely related to the chemical composition of the specimens before the heat treatment.

A comparative analysis of the structure of the CoCrMo alloys obtained at a minimum (T = 1830 K for 20 min) and at a maximum EBMR operating temperature (T = 1860 K for 20 min) after the heat treatment (Figure 3e,h) indicates that with the increase in the degree of removal of the alloying elements (Mn, Fe, and Si) and the enriching of the alloy with cobalt (Table 1), the size of the grains increases (from ~50 μm to ~200 μm) as the amount of inter-dendritic precipitates significantly decreases.

After the alloy’s heat treatment (Co-02-ann), the dendritic structure is broken. The formation of a finer-grained structure in the Co-02-ann sample is most likely due to the higher Si content in the sample and formation of Co_1.8_Cr(Mo,Si) phase, which can be obtained after the eutectoid decomposition of an ε-phase → Co_3_Cr+Co_2_Cr at T < 973 K in the presence of Mo and Si [14,23,26,27].

### 3.2. Analysis of Hardness of CoCrMo Alloys Recycled by EBMR

One of the main parameters determining the practical applicability of CoCrMo alloys in the dental industry is their hardness. From a practical point of view, it is important to determine the influence of the melting parameters (refining temperature and retention time) on the hardness of the ingots obtained after recycling by EBMR. The hardness of the initial CoCrMo alloy and of the ingots obtained in the electron beam furnace was measured during the research. The melting conditions and chemical compositions of the ingots are given in Table 1. The melt obtained is formed in a copper water-cooled crucible.

The influence of load 0.98 N, 1.96 N, 2.94 N, 4.9 N, and 9.8 N on the hardness of the resulting ingots is determined. The hardness is measured using the Vickers method, with nine measurements made at each load. The calculated mean hardness values and standard deviations are given in Table 2. The analysis of the results shows that as the load increases, the hardness of the alloy gradually decreases, then stabilizes and reaches an almost constant value.

**Figure 4:**
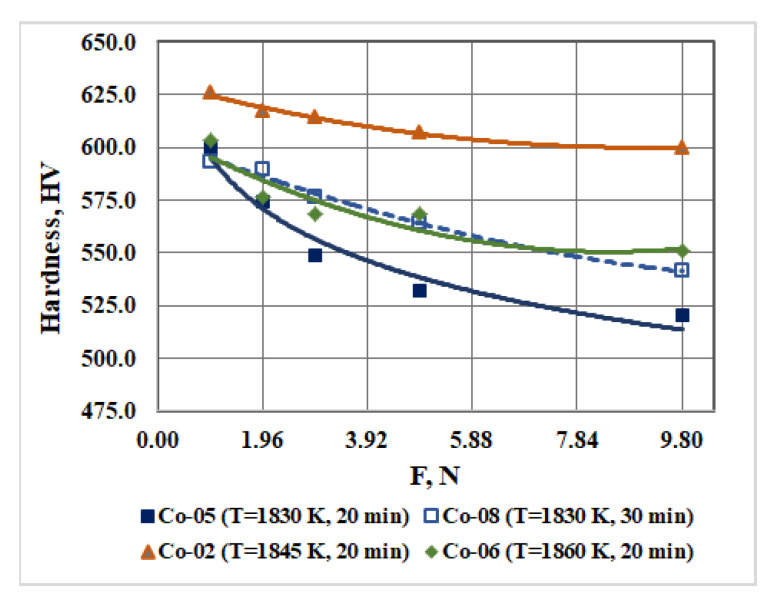
Influence of the EBMR technological parameters on the hardness of the obtained CoCrMo samples under different loads.

**Figure 5:**
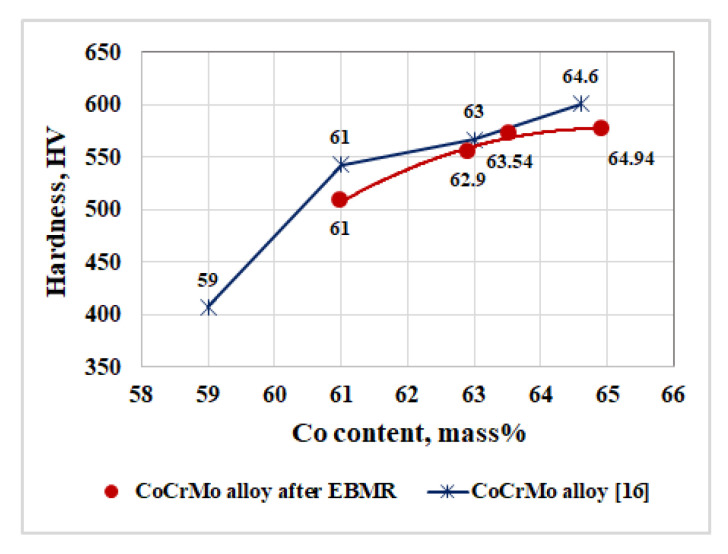
Influence of the cobalt content on the hardness of the produced CoCrMo specimens.

The influence of the technological parameters of EBMR of the technogenic CoCrMo alloy and the cobalt content on the hardness of the resulting CoCrMo ingots under different loads is shown in Figure 4 and Figure 5.

It can be seen that at refining temperatures of 1830 K and 1860 K for the same retention time (τ = 20 min), the hardness of the resulting ingots (measured at the highest load 9.8 N) increases from 520.3 ± 15.3 HV to 550.8 ± 21.9 HV. The highest hardness (600.0 ± 14.0 HV) is measured for the Co-02 ingot obtained at a refining temperature of 1845 K and a residence time of 20 min. This can be explained by the eutectoid decomposition of the ε-phase (ε → Co_3_Cr + Co_2_Cr) and the formation of finer grains [14,23,26,27].

The same figure (Figure 4) shows the dependence of the hardness (as a function of the load) for a CoCrMo alloy obtained at 1830 K for a longer refining time τ = 30 min. The measured hardness is 541.2 ± 12.6 HV (at the highest load 9.8 N) and it is slightly higher than the hardness of the alloy obtained at the same refining temperature for τ = 20 min. In this case, the higher hardness can be explained by the higher Co content in the alloy (Table 1).

The influence of the cobalt content on the hardness of CoCrMo alloys is presented in Figure 5. Figure 5 also shows the dependence presented in [16], where the hardness of different types of commercial CoCrMo alloys, used in medicine, has been measured. A correlation between the cobalt contents in the alloy and its hardness has been found. A similar finding is made in [4]. The base metal that determines the mechanical properties such as hardness, strength, and wear resistance of CoCrMo alloys is cobalt [4,16].

### 3.3. Analysis of Hardness of Heat-Treated CoCrMo Ingots Recycled by EBMR

The hardness after the heat treatment of: the starting CoCrMo alloy (initial alloy-ann) and the ingots obtained after recycling by EBMR (Co-05-ann, Co-08-ann, Co-02-ann, Co-06-ann) is also investigated. The mean hardness values measured at different loads and the calculated standard deviations of the heat-treated CoCrMo ingots are given in Table 3.

**Figure 6:**
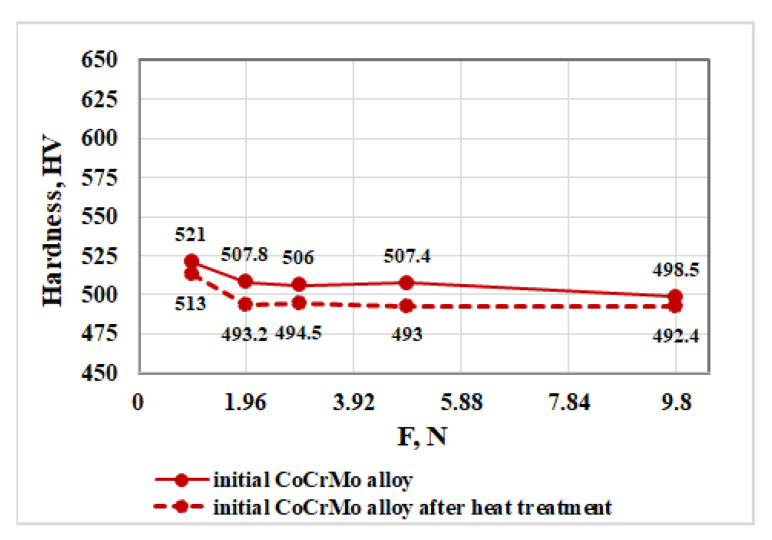
Influence of load on the hardness of the initial CoCrMo alloy before and after heat treatment.

Figure 6 shows the hardness values of the initial CoCrMo alloy obtained at different loads before and after the heat treatment.

The analysis of the resulting dependences (the hardness as a function of the load) shows that after the heat treatment of the alloy, its hardness decreases slightly compared to the initial material. The average hardness values for loads higher than 1.96 N remain almost constant and are 504.9 HV and 493.3 HV before and after the heat treatment, respectively.

**Figure 7:**
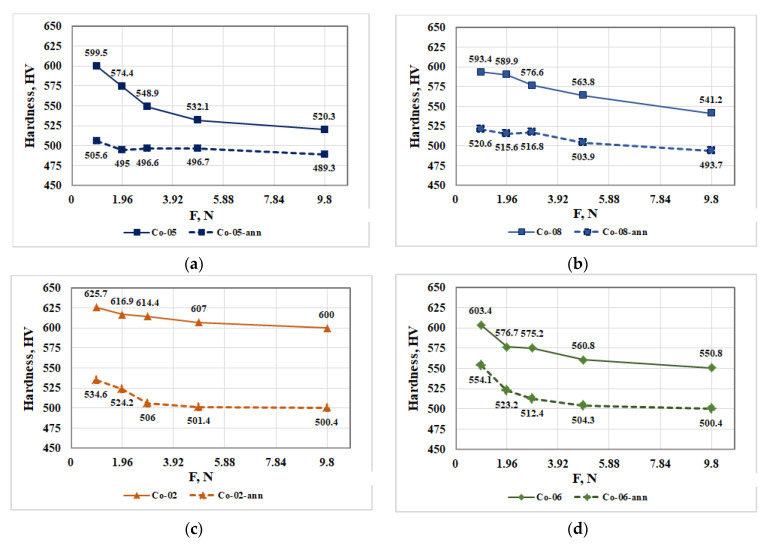
Influence of load on the hardness of CoCrMo specimens obtained after recycling by EBMR under different processing conditions (solid line) and after their heat treatment (dashed line): (**a**) Co-05 and Co-05-ann; (**b**) Co-08 and Co-08-ann; (**c**) Co-02 and Co-02-ann; (**d**) Co-06 and Co-06-ann.

The influence of load on the hardness of CoCrMo alloys recycled by EBMR and after their heat treatment is shown in Figure 7.

The analysis of the resulting dependences (Figure 7) shows that after the heat treatment of the ingots, recycled by EBMR, they have a significantly lower hardness compared to the samples before their heat treatment. This fact shows that significant phase transitions occur in the alloy during the heat treatment, which affect its hardness.

Figure 7 shows that in all studied samples, an increase in the load leads to a decrease in the hardness of the CoCrMo alloy and at a load higher than 2.94 N, it remains almost constant. It can be seen that regardless of the EBMR conditions for recycling of technogenic CoCrMo alloy, after the heat treatment, the hardness of the alloy varies in much narrower boundaries (Figure 7). The higher hardness values of the alloys recycled by EBMR can be explained by the higher cooling rate (water-cooled crucible) compared to the furnace cooling rate, applied after the heat treatment.

**Figure 8:**
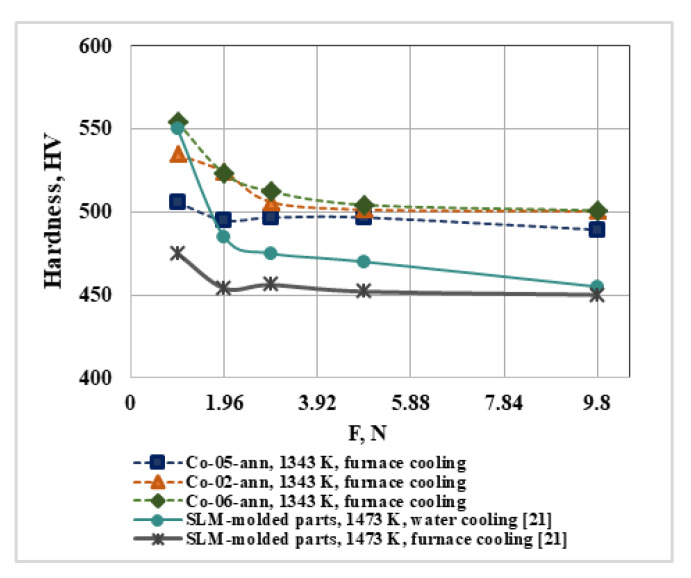
Influence of the conditions of heat treatment and cooling on the hardness of CoCrMo alloys under different loads.

The microstructure and, respectively, the mechanical properties of CoCrMo alloys, are determined by the speed of two main processes—cooling and solidification [28]. Figure 8 shows the hardness of ingots (Co-05-ann, Co-02-ann, and Co-06-ann) measured at different loads after the heat treatment, cooled in a muffle furnace in an argon atmosphere. The average hardness values after a load of 2.94 N are 494.2 HV, 502.6 HV, and 505.9 HV, respectively.

The same figure (Figure 8) compares the hardness values of the CoCrMo alloy measured in [21]. The chemical composition of the alloy, manufactured by PBF-LB [21], in mass% is: 62.44 Co, 26.27 Cr, 9.89 Mo, 0.38 Si, 0.70 Mn, and 0.25 Fe and it is close to that of the studied CoCrMo alloys. The hardness values measured of SLM parts heat-treated at 1473 K for 1 h with furnace cooling and for 2 h with water cooling are ~455 HV and ~486 HV, respectively [21].

Therefore, regardless of the different conditions for obtaining the CoCrMo alloy, the necessary hardness can be achieved through appropriate heat treatment and cooling rate.

## 4. Conclusions

Based on the conducted research regarding the impact of different electron beam melting parameters (temperature and residence time) and the heat treatment (under the same conditions) of ingots recycled by EBMR on the hardness of the CoCrMo alloys, the following conclusions can be made:

- As the e-beam refining temperature increases, at the same time of retention, the hardness of the recycled CoCrMo alloys, measured at the highest load of 9.8 N, increases from 520.3 HV to 550.8 HV. Extension of the residence time leads to an increase in the hardness of the alloy. The highest hardness (600 HV) is measured in the alloy recycled at 1845 K and a retention time of 20 min. This is due to the smaller crystal structure of the resulting alloy and the higher cobalt content.
- It has been found that regardless of the EBMR processing conditions, after the heat treatment, the hardness of the ingots varies within very narrow limits (from 494.2 HV to 505.9 HV) and is significantly lower than the hardness of the samples before their heat treatment.
- It has been found that the heat treatment process leads to considerable changes in the microstructure of the CoCrMo alloys recycled by EBMR. With the increase of the e-beam refining temperature, after the heat treatment, the grain size increases from ~50 to ~200 μm. The grains’ shape indicates an incomplete phase transition from γ-fcc to ε-hcp phase and this leads to a slight increase in the hardness of the alloy.
- It has been confirmed that the base metal that determines the hardness of CoCrMo alloys is cobalt. As the content of this metal in the alloy increases, its hardness also increases.

Through appropriate heat treatment, desired hardness of CoCrMo alloys recycled from a technogenic material (waste from the dental technology) by EBMR can be achieved.

## Figures and Tables

**Table 1:** Process parameters and chemical compositions (mass %) of the specimens before and after EBMR.

Sample	Parameter		Concentration of Basic Elements			Concentration of Other Elements					
	T, K	τ, min	Co	Cr	Mo	Fe	Mn	Nb	W	Si	Others
Co-0	Initial alloy		61	31.22	4.78	0.65	0.43	0.32	0.38	1.09	0.13
Co-05	1830	20	62.92	30.41	4.90	0.31	0.0	0.31	0.36	0.67	0.12
Co-08	1830	30	63.54	29.79	4.99	0.27	0.0	0.31	0.36	0.62	0.12
Co-02	1845	20	63.99	29.35	5.05	0.29	0.0	0.31	0.36	0.56	0.09
Co-06	1860	20	64.94	28.79	5.06	0.12	0.0	0.31	0.35	0.38	0.05

**Table 2:** Hardness (HV) and standard deviation (STD) of CoCrMo alloys recycled by EBMR.

Sample	HV ± STD of CoCrMo Alloys under Different Loads				
	0.98 N	1.96 N	2.94 N	4.9 N	9.8 N
Initial alloy	521.0 ± 18.1	507.8 ± 15.7	506.0 ± 22.7	507.4 ± 6.5	498.5 ± 9.8
Co-05	599.5 ± 10.6	574.4 ± 17.4	548.9 ± 21.6	532.1 ± 12.9	520.3 ± 15.3
Co-08	593.4 ± 16.4	589.9 ± 15.3	576.6 ± 7.0	563.8 ± 15.0	541.2 ± 12.6
Co-02	625.7 ± 15.4	616.9 ± 17.1	614.4 ± 12.1	607.0 ± 14.1	600.0 ± 14.0
Co-06	603.4 ± 28.6	576.7 ± 23.3	575.2 ± 29.3	577.0 ± 19.3	550.8 ± 21.9

**Table 3:** Hardness (HV) and standard deviation (STD) of the initial CoCrMo alloy and recycled alloy specimens after heat treatment.

Sample	HV ± STD of CoCrMo Alloys under Different Loads				
	0.98	1.96	2.94	4.9	9.8
Initial alloy-ann	513.0 ± 18.0	493.2 ± 11.6	494.5 ± 9.2	493.0 ± 16.3	492.4 ± 14.4
Co-05-ann	505.6 ± 9.5	495.0 ± 11.9	496.6 ± 8.9	496.7 ± 4.4	489.3 ± 11.1
Co-08-ann	520.6 ± 12.0	515.6 ± 11.1	516.8 ± 15.0	503.9 ± 7.7	493.7 ± 12.8
Co-02-ann	534.6 ± 18.2	524.2 ± 11.4	506.0 ± 8.6	501.4 ± 6.0	500.4 ± 13.0
Co-06-ann	554.1 ± 7.8	523.2 ± 11.9	512.4 ± 8.9	504.3 ± 12.7	500.9 ± 10.1

## Data Availability

Not applicable.
